# Endothelial Nitric Oxide Synthase *T-786C* Mutation, Prothrombin Gene Mutation (*G-20210-A*) and Protein S Deficiency Could Lead to Myocardial Infarction in a Very Young Male Adult

**DOI:** 10.3889/oamjms.2016.014

**Published:** 2016-01-29

**Authors:** Milka Klincheva, Elena Ambarkova Vilarova, Tanja Angjusheva, Ivan Milev, Enver Idoski, Zan Mitrev

**Affiliations:** *Special Hospital for Surgery Diseases “Filip II”, Skopje, Republic of Macedonia*

**Keywords:** (eNOS) *T-786C* mutation, prothrombin gene mutation (*G-20210-A*), protein S deficiency, myocardial infarction, young male

## Abstract

**INTRODUCTION:**

Myocardial infarction is a rare medical event in young people. The main reasons include congenital coronary abnormalities, coronary artery spasm, and coronary thrombosis due to hypercoagulable states (hereditary and acquired).

**AIM:**

We present a case of a young male adult with myocardial infarction caused by a combination of gene mutations and anticoagulation protein deficiency.

**CASE PRESENTATION:**

A 19 years old young man was admitted to our hospital complaining of chest pain during the last two weeks. The patient did not have any known cardiovascular risk factors, except a positive family anamnesis. Subacute inferior nonST segment myocardial infarction was diagnosed according to the patient’s history, electrocardiographic and laboratory findings. Coronary angiography revealed suboclusive thrombus in the proximal, medial and distal part of the right coronary artery (TIMI 2). Percutaneous coronary intervention was performed. Anticoagulant and antiagregant therapy (heparin, acetilsalicilic acid and clopidogrel) according to protocol was started. The hospital stay was uneventful. Homozygous endothelial nitric oxid synthase (eNOS) *T-786-C* mutation, heterozygote prothrombin gene mutation (*G-20210-A*), and protein S deficiency were verified from the thrombophilia testing. Other trombophilic tests were normal. Three months after discharge from hospital another coronary angiography was performed. It revealed normal coronary arteries. Four years after the attack, the patient is free of symptoms and another cardiovascular event.

**CONCLUSION:**

Combination of genetic mutations and anticoagulation protein deficiency could be a reasonable cause for myocardial infarction in a very young male adult without any other cardiovascular risk factors.

## Introduction

Acquired or hereditary hypercoagulability or thrombophilia may be defined as a tendency to venous or arterial thrombosis. Acquired hypercoagulability leading to arterial thrombosis is seen in every day surgical practice, while the genetic predisposition for development of arterial occlusive disease is still inconclusive. Past, as well as more recent publications suggest a correlation between the existence of particular genetic mutations and arterial thrombosis [1]. There is clear evidence that hyperhomocysteinaemia [2] and the antiphospholipid antibody syndrome [3] are a genetic predisposition to arterial thrombosis, but the role of prothrombin gene *G20210A* variant mutation and the factor V Leiden deficit is still unclear [4]. It seems that genetic mutations are particularly important for young patients and women since the data suggest that they increase the risk of myocardial infarction and ischemic stroke [5, 6].

Three to ten percent of all myocardial infarctions of young people occur due to non atherosclerotic disease such as congenital coronary artery anomalies, myocardial bridging, coronary artery dissection, septic vegetations, coronary artery aneurysms, hypercoagulable states (hereditary and acquired) [7, 8] and drug abuse.

We present a case of a young adult with subacute myocardial infarction, without the traditional coronary artery disease risk factors, and with a combination of gene mutations and anticoagulant protein deficiency.

## Case Presentation

**Figure 1:**
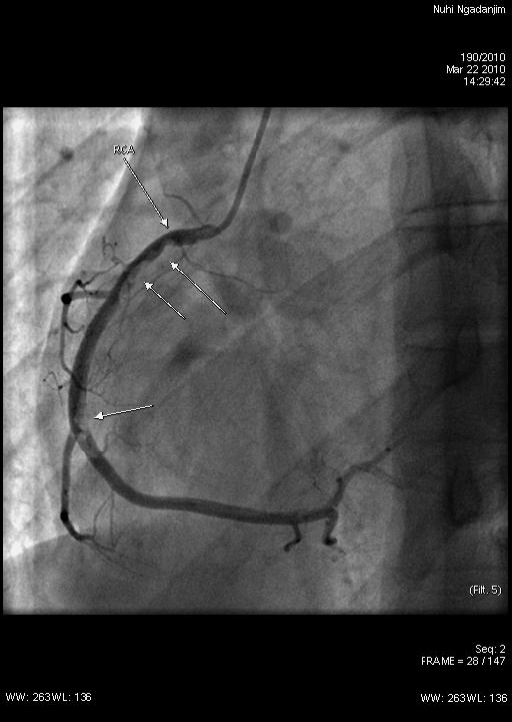
*Coronary angiography. Sub occlusive thrombus in the proximal, medial and distal part of the right coronary artery (TIMI 2)*.

A 19 year old young man was admitted to our hospital complaining of chest pain during the previous two weeks. He was not obese; he was an active athlete, and a nonsmoker. He does not consume alcohol regularly nor does he abuse drugs. The patient had no hypertension, hyperlipidemia or diabetes mellitus. He had no prior history of cardiovascular events. He has a positive family anamnesis for coronary artery disease. Electrocardiography showed sinus rhythm (HR 55/min), normal cardiac axis, 2 mm Q waves in D3, AVF, T wave negative in D2, D3, AVF, no ST segment elevation. Transthoracic echocardiography revealed hypokinesia of the inferior wall with preserved systolic function of the left ventricle. Laboratory analysis showed increased levels of the cardiac enzymes: creatine kinase 411 U/L (32-211 U/L), creatine kinase MB 8.8 ng/ml (0-15 ng/ml), troponin 16 ng/ml (0.78 ng/ml), and lactate dehydrogenase 947 U/l (208-378 U/l). Subacute inferior nonST segment myocardial infarction was diagnosed according to the patient’s history, electrocardiographic and laboratory findings [9]. Coronary angiography was performed. It revealed subocclusive thrombus in the proximal, medial and distal part of the right coronary artery (TIMI 2) (Fig. 1).

The other coronary arteries had no stenosis. Anticoagulant and antiagregant therapy (heparin, acetilsalicilic acid and clopidogrel) was started according to protocol [9]. The hospital stay was uneventful. After discharge, blood was drawn according to the guidelines for thrombophilia tests and sent for thrombophilic testing.

Homozygous endothelial nitric oxid synthase *(eNOS) T-786C* mutation, heterozygote prothrombin gene mutation (*G-20210-A*), and protein S deficiency were verified from the thrombophilia testing. Protein S deficiency activity was estimated at 10% (reference range 60-150%).

Deficit of antithrombin III, protein C and presence of antiphospholipid antibodies were excluded. There were no genetic mutations for factor V Leiden, genetic variants of the beta fibrinogen gene, gene for *PAI-1*, polymorphism in human platelet antigen (*HPA1*) (*1a/1a*), *MTHFR C677T* gene mutation, high levels of apolipoprotein B and factor XIII.

We recommended dual antiagregant therapy (acetilsalicilic acid a 100 mg plus clopidogrel a 75 mg per day) during one year after the myocardial infarction. After that, we recommended lifelong treatment with acetilsalicilic acid tablets.

**Figure 2:**
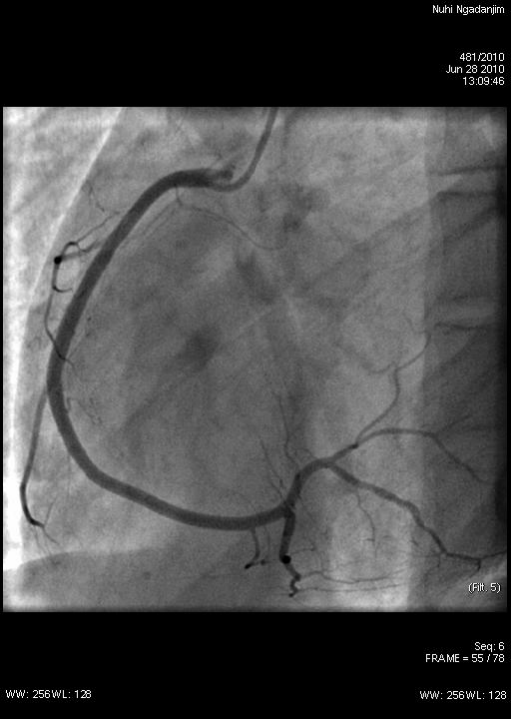
*Coronary angiography. Normal flow through the right coronary artery*.

Another coronary angiography was performed three months after discharge from hospital. It revealed normal coronary arteries (Fig. 2).

During 4 years follow up the patient was asymptomatic, without another acute coronary event.

## Discussion

In our paper we present a case of a very young male patient with subacute myocardial infarction due to thrombosis of the right coronary artery. After excluding the conventional cardiovascular risk factors, thrombophilic tests were performed and revealed homozygous endothelial nitric oxid synthase *(eNOS) T-786C* mutation, heterozygote prothrombin gene mutation (*G-20210-A*), and protein S deficiency.

Arterial thrombosis is a multi-factorial condition, where most of the risk factors do not overlap with those for venous thrombosis. An increased risk for arterial cardiovascular diseases by heritable thrombophilia could not be established firmly. The treatment and secondary prevention should relate to the established cardiovascular risk factors. Testing for heritable thrombophilia is not recommended, although there are many studies that confirm the coexistence between genetic mutation and arterial thrombosis.

Nitric oxide is produced by endothelium and plays an important role as a smooth muscle relaxant in maintaining vascular tone as well as inhibiting activation and aggregation of platelets [10]. Endothelial nitric oxid synthase catalyses the synthesis of nitric oxide. Presence of a *T-786-C* mutation in the *eNOS* gene results in decreased synthesis of nitric oxide and confers a greater risk for coronary artery spasm [11], ulcerative lesions in internal carotid artery [12]. Homozygous endothelial nitric oxide synthase *(eNOS) T-786-C* mutation is known as an independed risk factor in combination with other known cardiovascular risk factors, such as cigarette smoking [13]. In addition to the clinical connection between *eNOS T-786* mutation and risk for arterial thrombosis and atherosclerosis, there is molecular evidence for changes in markers of oxidative stress in high risk patients [14]. The presence of the *T-786* gene mutation in our patient, without other cardiovascular risk factors, but in combination with other gene mutations, suggests that this gene mutation is a potential cause for arterial thrombosis in such a constellation.

Prothrombin is a precursor of thrombin, acting as a pro-coagulant, via platelet activation and the generation of fibrin and factors Va, VIIIa, and XIIIa, and subsequently as an anticoagulant, by activating protein C. The prothrombin gene mutation is associated with elevated prothrombin levels, which is a genetically determined trait that increases the thrombosis risk [15]. About 1% to 2% of the general Caucasian population is heterozygous for the prothrombin gene which increases the risk of developing blood clots 2 to 5 times. Homozygous people have an even greater risk. Large number of studies demonstrated an association between the prothrombin *G20210A* mutation and venous thromboembolism. Recently, one study demonstrated a significant influence of prothrombin gene polymorphisms on myocardial perfusion [15]. The prothrombin *G20210A* mutation might play role in arterial thrombosis especially in young people [16] with other known cardiovascular risk factors.

Protein S, a vitamin K dependent physiological anticoagulant, acts as a nonenzymatic cofactor to activated protein C in the proteolytic degradation of a factor Va and factor VIIIa. Protein S deficiency may be hereditary or acquired (due to hepatic disorders or a vitamin K deficiency). Hereditary protein S deficiency represents an autosomal dominant trait and manifestations of thrombosis are observed in both heterozygous and homozygous individuals. Protein S deficiency usually manifests clinically as venous thromboembolism and the absolute risk of thrombosis in patients with protein S deficiency has been estimated to be 8.5 times higher than in individuals with no defect [17]. There are studies that suggest an association between arterial thromboses (stroke, heart attack) in patients with protein S deficiency, but its role in arterial disease is still being explored [18]. In patients younger than 55 years of age protein S deficiency should be considered as atherothrombotic risk factor [19].

Venous and arterial thromboses are complex genetic traits, with various gene-gene and gene-environment interactions. A number of studies have evaluated and confirmed that the coinheritance of two or more defects increases the appearance of thrombosis [20].

In spite of the fact that opinions about protein S deficiency and prothrombin *G20210A* mutation and their role in the appearance of arterial thrombosis are divided, the combination with other genetic defects like *T-786-C* mutation in the *eNOS* gene, may enhance the probability of arterial thrombosis like myocardial infarction, especially in young individuals without any other risk factors. In these clinical cases, testing for coagulation disorders should be performed in order to recommend a long term therapy which could prevent any other thrombotic events.

In conclusion, the combination of genetic mutations and anticoagulation protein S deficiency could be a reasonable cause for myocardial infarction in a very young male adult without any other cardiovascular risk factors. This case suggests that in premature coronary artery disease thrombophilia tests are justified. In addition to its scientific value, genetic tests could be a solid basis for further therapy recommendation.
